# Source and Health Risk Assessment of Heavy Metals in Soil–Ginger System in the Jing River Basin of Shandong Province, North China

**DOI:** 10.3390/ijerph18136749

**Published:** 2021-06-23

**Authors:** Songtao Wang, Zongjun Gao, Yuqi Zhang, Hairui Zhang, Zhen Wu, Bing Jiang, Yang Liu, Hongzhi Dong

**Affiliations:** 1 The Fourth Geological Brigade of Shandong Provincial Bureau of Geology and Mineral Resources, Weifang 261021, China; wangsongtao@sddksy.com (S.W.); zhanghairui@sddksy.com (H.Z.); wuzhen@sddksy.com (Z.W.); jiangbing@sddksy.com (B.J.); liuyang@sddksy.com (Y.L.); 2 Key Laboratory of Coastal Zone Geological Environment Protection of Shandong Geology and Mineral Exploration and Development Bureau, Weifang 261021, China; 3 College of Earth Science and Engineering, Shandong University of Science and Technology, Qingdao 266590, China; zongjungao1964@163.com (Z.G.); dhz@sdust.edu.cn (H.D.)

**Keywords:** heavy metals, soil, ginger, health risk assessment, Jing River Basin

## Abstract

This study investigated the characteristics and sources of heavy metals in a soil–ginger system and assessed their health risks. To this end, 321 topsoil samples and eight soil samples from a soil profile, and 18 ginger samples with root–soil were collected from a ginger-planting area in the Jing River Basin. The average concentration of heavy metals in the topsoil followed the order: Cr > Zn > Pb > Ni > Cu > As > Cd > Hg. In the soil profile, at depths greater than 80 cm, the contents of Cr, Ni, and Zn tended to increase with depth, which may be related to the parent materials, whereas As and Cu contents showed little change. In contrast, Pb content decreased sharply from top to bottom, which may be attributable to external environmental and anthropogenic factors. Multivariate statistical analysis showed that Cr, Ni, Cu, Zn, and Cd contents in soil are affected by natural sources, Pb and As contents are significantly affected by human activities, and Hg content is affected by farmland irrigation. Combined results of the single pollution index (*P_i_*), geo-accumulation index (*I_geo_*), and potential ecological risk assessment (*E_i_* and *RI*) suggest that soil in the study area is generally not polluted by heavy metals. In ginger, Zn content was the highest (2.36 mg/kg) and Hg content was the lowest (0.0015 mg/kg). Based on the bioconcentration factor, Cd and Zn have high potential for enrichment in ginger. With reference to the limit of heavy metals in tubers, Cr content in ginger exceeds the standard in the study area. Although Cr does not accumulate in ginger, Cr enrichment in soil significantly increases the risk of excessive Cr content in ginger.

## 1. Introduction

Heavy metals, which generally include As, Cr, Cd, Pb, and other biotoxic elements, are persistent pollutants that continuously accumulate in the environment. These pollutants accumulate in soil through various sources, such as industrial activities, fertilization and irrigation, and rock weathering [1]. Heavy metals in soil can be taken up by plants and then reach the human body through the food chain, seriously threatening human life and health [2,3,4]. With their long biological half-lives, nonbiodegradability, and ability to chronically accumulate in different parts of the body, such as the kidneys and liver, heavy metals are extremely harmful [5]. In appropriate amounts, trace metals, such as Cu, Zn, Fe, Mn, and Cr, play an important role in enzyme structuring and the synthesis of hemoglobin and vitamins, but excess contents of these metals can be harmful. In contrast, heavy metals such as Cd and Pb are toxic, even at low concentrations [6]. Therefore, it is of great significance to identify the characteristics, distribution, and sources of heavy metals in soil–crop systems and conduct associated health risk assessments.

Determining the source of heavy metals is key to preventing and controlling heavy metal pollution. Multivariate statistical analyses have been widely used to trace the source of heavy metals, and factor analysis (FA) and hierarchical clustering analysis (HCA) have commonly been employed [7,8,9,10]. FA is advantageous in that it uses a small number of variables to explain complex problems. Using R-type HCA, variables with large differences can be separated and similar variables can be clustered. Huang et al. [11] quantified the contribution of each heavy metal source using FA combined with absolute principal component scores/multiple linear regression (APCS-MLR), considering that natural sources contribute the most to Cr, Ni, and Cu contents, agricultural activities contribute the most to Zn, Cd, and Pb contents, industrial emissions contribute the most to As content, and coal burning contributes the most to Hg content, with other possible unknown sources, such as traffic or domestic sewage. Sun [12] performed HCA on heavy metal contents in roots, stems, and leaves of tea trees in Tieguanyin Tea Garden in the southeast of Fujian Province and suggested that the results of HCA are consistent with those of FA.

Over the past few decades, environmental scientists have developed a number of methods for assessing heavy metal pollution. Classical index evaluation methods include single pollution index (*P_i_*) [13], geo-accumulation index (*I_geo_*) [14], and potential ecological risk assessment method (*E_i_* and *RI*) [15]. The *P_i_* method reflects the pollution of heavy metals in soil by comparing observed values with standard limit values. The *I_geo_* method is based on the geochemical background values of heavy metals, using the logarithmic evaluation results, and considering the correction coefficient of changes in background values, which may be caused by diagenetic factors; this approach is more intuitive for evaluating samples with relatively high contamination levels. The Ei and RI methods eliminate differences attributable to background values of elements and comprehensively reflect the impact of heavy metals on the ecological environment from the three major aspects of ecology, environment, and toxicology; accordingly, these methods have a wide range of applicability [16,17,18,19,20,21,22].

Ginger is a common spice in daily food and is also a known medicinal plant [23]. Heavy metals in soil significantly affect the growth of ginger. Li et al. [24] suggested that a Pb content of 250 mg/kg in soil slightly promoted the growth of ginger, but Pb contents of 500–1000 mg/kg significantly inhibited its growth. As ginger is directly ingested by humans, some heavy and trace metals with potential long-term health risks may be indirectly taken up. Therefore, the study of heavy metals in ginger is of great significance. A standardized production base of ginger has been established in villages along the Jing River in Shandong Province. With its thin rind, bright yellow color, and strong flavor, ginger from this region has been sold not only within China, but also to more than 10 countries.

In this study, the contents of Cr, Ni, Cu, Zn, Cd, Pb, As, and Hg in a soil–ginger system in the Jing River Basin were analyzed with the following aims: (1) to trace the potential sources of heavy metals; (2) to assess the health risks of heavy metals in the soil–ginger system. The results of this study can provide insights for the prevention and control of local soil heavy metal pollution, and scientific planting of ginger.

## 2. Materials and Methods

### 2.1. Study Area

**Figure 1:**
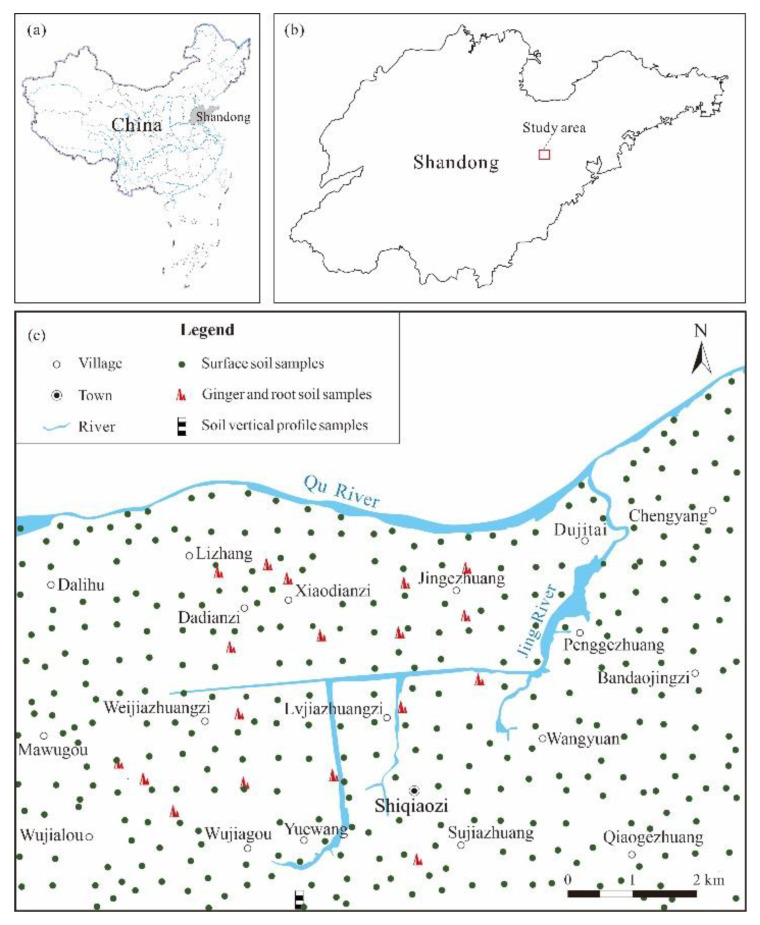
Location map of the study area (**a**) Location of Shandong Province (**b**) Location of the study area (**c**) Sampling location.

The study area, located in Weifang City, Shandong Province, is a standardized ginger planting area established along the Jing River, the main tributary of the Qu River (Figure 1). It is located in the north temperate monsoon climate zone, with four distinct seasons. The mean annual precipitation is approximately 740 mm, of which the months of June, July, and August account for approximately 60–70%. In the study area, brown soil with high clay content and low organic matter is widely distributed in most areas west of Shiqiaozi Town. In the area east of Shiqiaozi Town, the soil type is leached cinnamon soil, and the parent material is mainly composed of residual deposits and pluvial alluvial deposits of limestone, sandstone, and shale. A small part of the area features neutral skeleton soil with darker color.

### 2.2. Data Source

Geochemical data of soil and ginger used in the present study were provided by the Fourth Geological Brigade of Shandong Provincial Bureau of Geology and Mineral Resources. The sampling site is located in the ginger-planting area of the Jing River Basin in Shandong Province. The land-use type is mainly cultivated land, with problems such as excessive irrigation. In addition, the development of machinery foundry, building materials, and other enterprises in the study area has improved the economic performance, but also significantly increased the amount of sewage discharge. This led to an increase in the content of heavy metals, some of which may have accumulated in crops, such as ginger.

In October 2018, 321 topsoil samples, 8 soil samples from a soil profile, and 18 ginger samples with root–soil were collected from the study area. Sampling points of surface soil were laid out in grids of 1 × 1 km, and the average sampling density of soil samples was 5.5 pieces/km^2^. The depth of the soil profile was 2 m; one sample was collected every 0.2 m within 0–1 m, and samples were collected at 1.3, 1.6, and 2.0 m within 1–2 m. Soil samples were air dried at 25 °C, broken with a wooden stick, and sieved through a 10-mesh sift. Ginger samples were collected in the peak harvest period. Sampling units of 0.1–0.2 hm^2^ were set, and 5–20 ginger samples were collected from each sampling unit. The samples were then mixed in equal amounts to form a mixed sample. The ginger samples were rinsed in the fresh state to remove adhered soil and contamination by fertilization and spraying of pesticides, and then dried at room temperature.

All samples were sent to the Experimental Testing Center of the Fourth Geological Brigade of Shandong Provincial Bureau of Geology and Mineral Resources for testing. Heavy metals were determined according to the Specification of Multi-purpose Regional Geochemical Survey (1:250,000) (DZ/T 0258-2014), Specification of Regional Ecogeochemistry Assessment (DZ/T 0289-2015), Technical Requirements for Analysis of Ecological Geochemical Evaluation Samples (Trial), and the Specification of Testing Quality Management for Geological Laboratories (DZ/T 0130.4-2006). For Cd analysis, samples were dissolved in HF, HNO_3_, and HClO_4_ to catch fluorine, and extracted with HNO_3_; the volume was metered and Cd was then determined by inductively coupled plasma mass spectrometry (ICP-MS). For Cu, Pb, Zn, Ni, and Cr, samples were processed using the powder pressure method and tested by X-ray fluorescence spectrometry (XRF). For As and Hg, samples were pretreated with aqua regia and tested by atomic fluorescence spectrometry (AFS). For pH, samples were immersed in distilled water without carbon dioxide and tested using the ion selective electrode (ISE) method.

To ensure strict quality assurance and control procedures, 4 national first-level reference materials (GBW) (internal quality control) and 2 external standard control samples (external quality control) were inserted for every 50 samples, and their accuracy and precision were 100% qualified. At the same time, 2 replicate samples were set for every 50 samples for repeatability inspection. The pass rate of each index was between 96.2% and 99.1% (>90%), which meets test quality requirements of the “Specification of Land Quality Geochemical Assessment” (DZ/T 0295-2016).

### 2.3. Data Analysis

The basic data were analyzed in Microsoft Excel, and the statistical analyses of soil physical and chemical properties and heavy metal contents were performed in SPSS 25 (Chicago, IL, USA). A diagram of heavy metal contents in the soil profile was drawn in Origin 7.5 (Northampton, MA, USA) to reflect their distribution.

FA is the most commonly used method of dimensionality reduction, which can be used to reduce the multi-element heavy metal dataset to 3–4 factors, and then determine the potential sources of heavy metals by determining the source of each factor. FA requires a strong correlation between the original variables, which is usually determined using the KMO test and Bartlett’s test. If the KMO test coefficient is greater than 0.5, and the *p* value (significance probability) of Bartlett’s Test is less than 0.05, the data is suitable for FA. R-type HCA was used to classify heavy metals, such that each category can represent certain characteristics, which can verify the results of FA. Correlation analysis was applied to describe the closeness between two variables, and then reveal the synergistic and antagonistic effects between heavy metals.

The bioconcentration factor (BCF) is the ratio of the content of a certain element in plants to the content of that element in soil (Equation (1)), which reflects the ability of the metal to migrate from the soil to the plant to a certain extent [25,26,27,28].

(1) $$BCF=C_{i-ginger}/C_{i-soil}$$

where *C_i-ginger_* is the measured concentration of the *i*th heavy metal in ginger; *C_i-soil_* is the measured concentration of heavy metal *i* in root–soil.

*P_i_*, *I_geo_*, *E_i_*, and *RI* are commonly used indices for the evaluation of soil heavy metal pollution [29,30]. The equations and degrees of these indices are shown in Table 1.

## 3. Results and Discussion

### 3.1. Soil Geochemistry

#### 3.1.1. Average Concentration of Heavy Metals

The pH value of surface soil in the ginger-planting area ranged from 4.24 to 7.72, showing weakly acidic–neutral soil. The average concentrations of heavy metals followed the order: Cr > Zn > Pb > Ni > Cu > As > Cd > Hg (Table 2). The coefficient of variance (CV) is an important parameter that reflects elemental distribution. The CV values of Cr, Zn, Pb, Ni, Cu, As, and Cd ranged from 0.12 to 0.37, indicating a relatively uniform distribution. In contrast, the CV value of Hg was 0.57, reflecting an uneven distribution. The contents of Cd, Zn, Ni, Cu, and As in soil were below the reference background values of Shandong. The contents of Cr, Hg, and Pb in the study area were relatively high at 1.08–1.09 times those of their averages in the province, reflecting relatively high levels of pollution.

**Figure 2:**
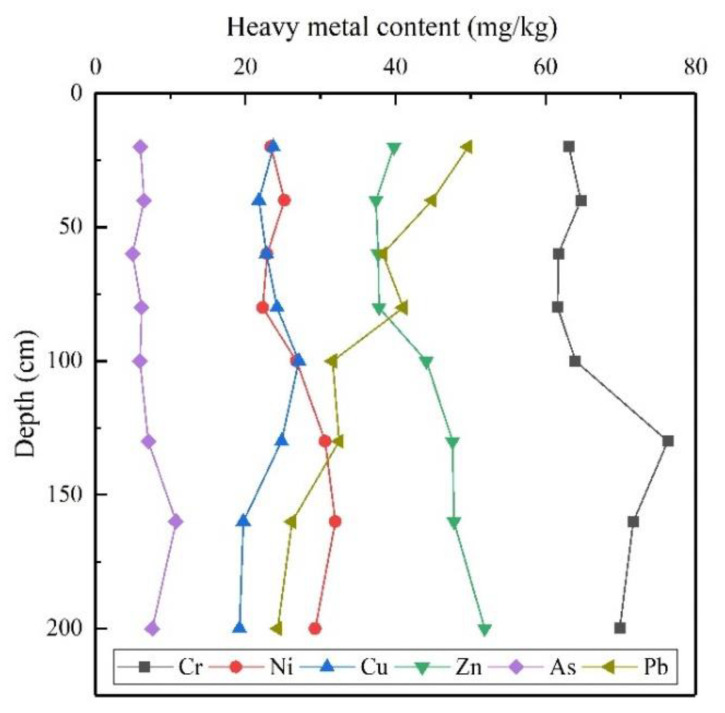
Contents of heavy metals (HMs) in soil profiles.

Many studies [31,32,33,34,35] have shown that metals such as Pb, Zn, and Cd can accumulate in the soil plowing layer and migrate vertically. In Figure 2, the contents of As and Cu showed little change with increasing depth. The contents of Cr, Ni, and Zn showed little change within a depth range of 0–80 cm, below which they tended to increase with depth, indicating the influence of the parent materials of soil. The content of Pb was concentrated in the surface layer of soil and decreased sharply from top to bottom, suggesting that it is mainly controlled by external environmental and anthropogenic factors.

#### 3.1.2. Source of Heavy Metals

The accumulation of heavy metals in soil poses a serious threat to the environment, agricultural production, and human health. Through absorption by crops, heavy metals enter the food chain, thereby threatening human health [2]. Increased concentrations of heavy metals in farmland are attributable not only to the parent material of soil, but also to human activities, such as mining, smelting, fossil fuel burning, and sewage irrigation [36,37].

The results of the KMO test (0.715) and Bartlett’s test (*p* < 0.05) revealed a good correlation between variables. Thus, they are suitable for FA (Table 3).

**Figure 3:**
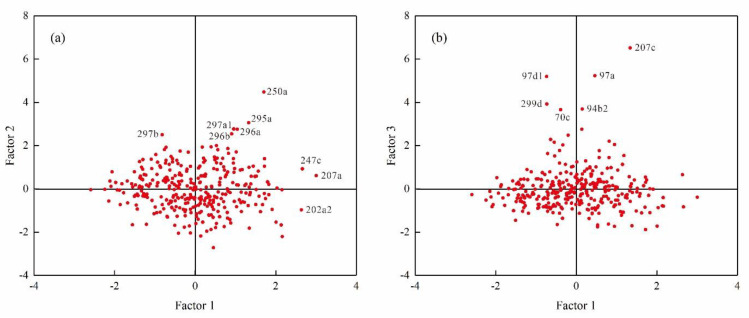
Scatter diagram of FA: (**a**) Factor 1 vs. Factor 2; (**b**) Factor 1 vs. Factor 3.

The results of FA show that the initial eigenvalues of the first three factors are higher than 1, with the cumulative contribution rate reaching 70% (Table 4). The contribution rate of factor 1 was 40.4%, for which Cr (0.8), Ni (0.86), Cu (0.76), Zn (0.78), and Cd (0.59) exhibited higher factor loadings. These five elements were all positively correlated with factor 1 (Table 5), indicating that they have similar distribution characteristics in soil. This indication is mainly manifested in samples 02a2, 207a, and 247c (Figure 3), with a relatively dispersed distribution, which is presumed to be affected by natural sources. The contribution rate of factor 2 was 15.8%, for which Pb (0.71) and As (0.54) exhibited higher factor loadings. As shown in Figure 3, samples 250a, 295a, 296a, 296b, 297a1, and 297b, which were sampled near the town, exhibited higher factor scores. In addition, Pb is a characteristic feature of coal burning [38]. Therefore, factor 2 is speculated to potentially reflect anthropogenic influences. The contribution rate of factor 3 was 13.6%, for which Hg (0.9) exhibited a higher factor loading. The factor scores of samples 70c, 94b2, 97a, 97d1, 207c, and 299d were higher (Figure 3). The Hg content (0.0081 mg/L) of farmland irrigation water near these points exceeded the standard. Thus, factor 3 is speculated to reflect the influence of farmland irrigation.

**Figure 4:**
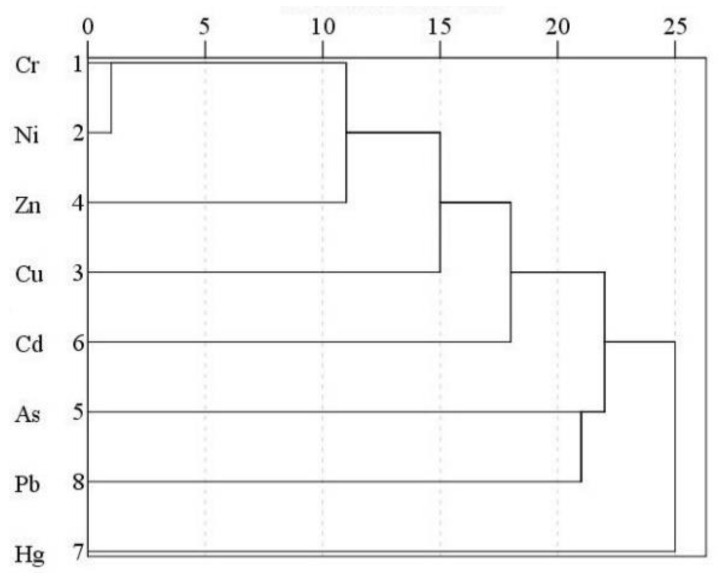
Dendrogram of HCA.

To further reveal the correlation between heavy metals, HCA was conducted on eight heavy metal elements in soil. The results are shown in Figure 4. Based on the results, the eight heavy metals can be divided into three clusters at a rescaled distance of 22. Cr, Ni, Cu, Zn, and Cd belong to cluster 1, Pb and As belong to cluster 2, and Hg belongs to cluster 3. This classification is basically consistent with the FA results.

### 3.2. Average Concentration of Heavy Metals in Ginger and Root–Soil

When the concentration of heavy metals in animals and plants exceeds a certain threshold, they cause harm. In particular, heavy metals enter the human body through the food chain and accumulate continuously, increasing the risk of cancer and other diseases. Therefore, it is important to study the content characteristics of heavy metals in crops. Such studies would contribute towards controlling the quality of crops, as well as characterizing the absorption intensity of heavy metals in soil by crops.

The average concentrations of heavy metals in ginger followed the order: Zn > Cu > Ni > Cr > Pb > As > Cd > Hg (Table 6). Hg was not detected in the samples. Half of the detection limit was used for statistics. The CV values of Zn and As were 0.26 and 0.31, respectively, indicating uniform distribution. The CV values of Cu, Ni, Pb, Cr, and Cd ranged from 0.41 to 0.74, indicating uneven distribution.

The average concentration of heavy metals in root–soil followed the order: Cr > Zn > Pb > Cu > Ni > As > Cd > Hg (Table 7). The CV values of Cr, Ni, As, Zn, Pb, Cu, and Hg were between 0.17 and 0.28, indicating relatively uniform distribution; the CV value of Cd was 0.55, suggesting uneven distribution.

Different heavy metals show different levels of enrichment in plants. As shown in Table 7, the *BCF* values followed the order: Cd > Zn > Hg > Cu > Ni > Cr > As > Pb. In general, Cd and Zn have greater potential for enrichment in ginger compared to Cr, As, Dand Pb. The *BCF* value of Cd was 53 times that of Pb. The chemical properties of heavy metals are one of the important factors affecting their migration and enrichment in the soil–ginger system.

The positive and negative correlations between elements in plants reveal their synergistic and antagonistic effects, respectively. Table 8 shows a significant positive correlation between Cr, Ni, and Cd in ginger, indicating that these three heavy metals co-operate with each other to promote absorption. No obvious correlation was observed among the other heavy metals.

### 3.3. Health Risk Assessment of Heavy Metals

#### 3.3.1. Pollution Assessment for Heavy Metal Pollution in Soil

**Figure 5:**
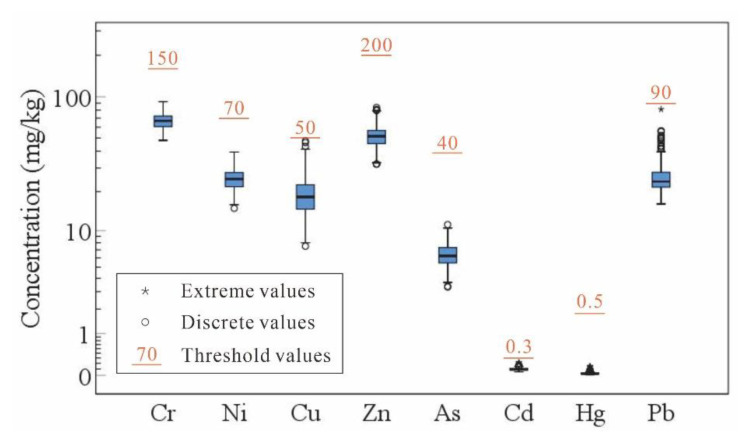
Box plot of heavy metal contents in surface soil.

The content of heavy metals in surface soil of the study area did not exceed the risk control standard for soil contamination of agricultural land (GB 15618-2018) (Figure 5). Therefore, surface soil of the study area meets the second-level national requirements for soil environmental quality. It is highly important to use appropriate methods for evaluating the degree of heavy metal pollution in soil to control ecological and environmental pollution. In this study, according to background values of heavy metals in the study area and risk screening values for soil contamination of agricultural land, the calculated *P_i_* values (0.07–0.45) were all below 0.7, and *I_geo_* values (from −0.46 to −1.01) were all below 0. Except for Hg (43.37), *E_i_* values (0.82–23.46) of the other heavy metals were all below 40. The calculated *RI* value (91.59) was less than 150 (Table 9). According to these results, there is basically no heavy metal pollution in the soil of the study area.

#### 3.3.2. Safety Assessment of Ginger

**Figure 6:**
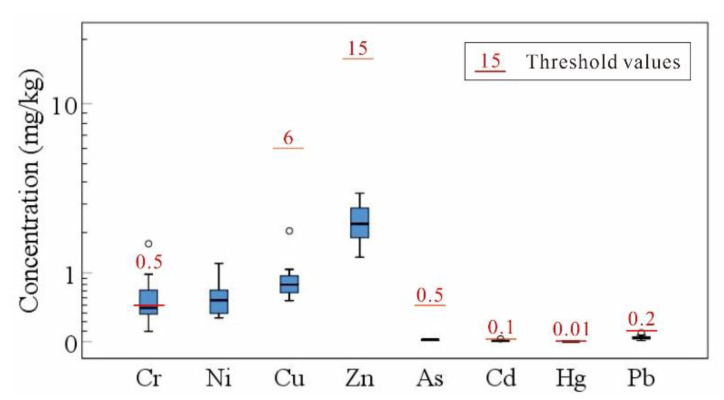
Box plot of heavy metal contents in ginger.

The heavy metal safety of ginger in the study area was assessed according to the limits of As, Cd, Cr, Hg, and Pb stipulated in the People’s Republic of China “National Food Safety Standard Contamination Limit in Food” (GB 2762-2017), and limits of Cu and Zn stipulated in the People’s Republic of China agricultural industry standards “Limits of Eight Elements in Cereals, Legume, Tubers and its Products” (NY 861-2004). The results showed that six ginger samples contained excessive Cr contents, with an exceeding rate of 33%. None of the other heavy metals exceeded the standard (Figure 6). Although previous analyses showed that the enrichment of Cr is weak in ginger, the content of Cr in soil was the highest among the eight heavy metals. Thus, the enrichment of Cr in soil significantly increases the risk of excessive Cr in ginger.

## 4. Conclusions

In this study, the characteristics, sources, and health risks of heavy metals in a soil–ginger system in the Jing River Basin were investigated. The content of Cr in surface soil was the highest, reaching 66.92 mg/kg, and the content of Hg was the lowest at 0.03 mg/kg. The CV value of Hg was 0.57, indicating a relatively uneven distribution. The CV values of the other heavy metals ranged from 0.12 to 0.37, indicating a relatively uniform distribution. In the soil profile, the contents of As and Cu showed little change, while those of Cr, Ni, Zn, and As increased with depth within a depth range of 80–200 cm. Pb was concentrated only in the surface layer of soil, which may be attributable to external environmental and anthropogenic factors. The results of factor analysis (FA) and hierarchical clustering analysis (HCA) showed that the contents of Cr, Ni, Cu, Zn, and Cd in soil may be affected by the parent materials of soil, those of Pb and As by human activities, and that of Hg by irrigation water. The calculated results of *P_i_* (0.07–0.45), *I_geo_* (from −0.46 to −1.01), *E_i_* (0.82–43.37), and *RI* (91.59) show that the soil in the study area is not polluted by heavy metals. Cd and Zn have potential for enrichment in ginger, with *BCF* values of 0.0687 and 0.0343, respectively. The BCF value of Cr (0.0066) was relatively small, but the Cr content in ginger exceeded the limit, possibly because Cr has the highest content in soil among the eight heavy metals.

## Figures and Tables

**Table 1:** Classification of *P_i_*, *I_geo_*, *E_i_* and *RI*.

Index	Equation		Category	Degree
Single pollution index (*P_i_*)	$P_{i}=C_{i}/C_{ti}$	(2)	*P_i_* < 0.7	Nonpollution (soil), clean (crop)
			0.7 ≤ *P_i_* < 1	Nonpollution (soil), slightly clean (crop)
			1 ≤ *P_i_* < 2	Slight pollution (soil), moderate pollution (crop)
			2 ≤ *P_i_* < 3	Moderate pollution (soil and crop)
			*P_i_* ≥ 3	Heavy pollution (soil and crop)
Geo-accumulation index (*I_geo_*)	$I_{geo}=\log_{2}(C_{i}/1.5C_{bi})$	(3)	*I_geo_* < 0	Nonpollution
			0 ≤ *I_geo_* < 1	Slight pollution
			1 ≤ *I_geo_* < 2	Moderate pollution
			2 ≤ *I_geo_* < 3	Moderate to heavy pollution
			3 ≤ *I_geo_* < 4	Heavy pollution
			4 ≤ *I_geo_* < 5	Heavy to extreme pollution
			*I_geo_* ≥ 5	Extreme pollution
Potential ecological risk assessment method (*E_i_* and *RI*)	$E_{i}=T_{i}\times C_{i}/C_{bi}$ $RI=\sum_{i=1}^{n}E_{i}$	(4) (5)	*E_i_* < 40; *RI* < 150	Low potential ecological risk
			40 ≤ *E_i_* < 80; 150 ≤ *RI* < 300	Moderate potential ecological risk
			80 ≤ *E_i_* < 160; 300 ≤ *RI* < 600	Considerable potential ecological risk
			160 ≤ *E_i_* < 320; *RI* ≥ 600	High potential ecological risk
			*E_i_* ≥ 320	Extreme potential ecological risk

*C_i_*: measured concentration of the *i*th heavy metal; *C_ti_*: the limit value of the *i*th heavy metal; *C_bi_*: background value of the *i*th heavy metal; *E_i_*: single-factor potential ecological risk index of the *i*th heavy metal; *T_i_*: toxicity coefficient of the *i*th heavy metal; *RI*: the comprehensive ecological risk of the *i*th heavy metal.

**Table 2:** Statistical characteristics of heavy metal contents in surface soil (mg/kg).

Data	Cr	Ni	Cu	Zn	As	Cd	Hg	Pb	pH
Mean	66.92	25.04	19.4	51.68	6.4	0.1	0.03	25.65	6.21
Minimum	48.1	14.9	7.5	32.1	3.32	0.06	0.01	16.1	4.24
Maximum	92.1	39.4	47.6	83.7	11.16	0.24	0.17	81.2	7.72
CV	0.12	0.18	0.37	0.18	0.21	0.25	0.57	0.28	0.1
Shandong background value	62	27.1	22.6	63.3	8.6	0.13	0.03	23.6	7.32
K	1.08	0.92	0.86	0.82	0.74	0.78	1.08	1.09	0.85

K: ratio of mean value to the background value in Shandong.

**Table 3:** Results of the KMO test and Bartlett’s test.

KMO and Bartlett’s Test		
Kaiser–Meyer–Olkin Measure of Sampling Adequacy.		0.715
Bartlett’s Test of Sphericity	Approx. Chi-Square	968.005
	df	28
	Sig.	0.000

**Table 4:** Explanation of total variance.

Component	Initial Eigenvalues			Extraction Sums of Squared Loadings		
	Total	% of Variance	Cumulative %	Total	% of Variance	Cumulative %
1	3.232	40.406	40.406	3.232	40.406	40.406
2	1.261	15.759	56.164	1.261	15.759	56.164
3	1.090	13.630	69.794	1.090	13.630	69.794
4	0.789	9.860	79.654			
5	0.720	9.000	88.653			
6	0.432	5.404	94.058			
7	0.354	4.421	98.479			
8	0.122	1.521	100.000			

**Table 5:** Component matrix.

Heavy Metal	Component		
	1	2	3
Cr	**0.804**	−0.457	−0.062
Ni	**0.860**	−0.284	−0.205
Cu	**0.755**	0.276	−0.185
Zn	**0.779**	−0.239	0.214
As	0.414	**0.544**	−0.122
Cd	**0.586**	0.132	0.350
Hg	0.103	0.137	**0.901**
Pb	0.382	**0.711**	−0.123

Bold data show higher factor loadings.

**Table 6:** Statistical characteristics of heavy metal contents in ginger (mg/kg).

Data	Cr	Ni	Cu	Zn	As	Cd	Hg	Pb
Sample number	18	18	18	18	18	18	18	18
Mean	0.52	0.55	0.84	2.36	0.02	0.0096	0.0015	0.04
Minimum	0.11	0.27	0.51	1.34	0.01	0.0025	0.0015	0.01
Maximum	1.68	1.20	2.05	3.46	0.03	0.0280	0.0015	0.09
CV	0.72	0.46	0.41	0.26	0.31	0.74	0.00	0.51
BCF	0.0066	0.0184	0.0267	0.0343	0.0024	0.0687	0.0335	0.0013

**Table 7:** Statistical characteristics of heavy metal contents in root–soil (mg/kg).

Data	Cr	Ni	Cu	Zn	As	Cd	Hg	Pb
Sample number	18	18	18	18	18	18	18	18
Mean	79.49	29.81	31.31	68.81	7.43	0.14	0.04	31.83
Minimum	57.90	19.70	23.30	44.80	5.50	0.07	0.02	25.10
Maximum	93.70	40.30	51.00	105.00	9.59	0.38	0.07	49.70
CV	0.12	0.17	0.22	0.20	0.17	0.55	0.28	0.21

**Table 8:** Correlation analysis of heavy metal contents in ginger.

Heavy Metal	Cr	Ni	Cu	Zn	As	Cd	Pb
Cr	1	0.790 **	0.197	0.362	0.114	0.498 *	0.331
Ni		1	0.356	0.091	−0.015	0.672 **	0.373
Cu			1	0.166	−0.271	0.331	0.238
Zn				1	0.172	0.268	0.119
As					1	0.044	0.050
Cd						1	0.162
Pb							1

** Correlation is significant at the 0.01 level (2-tailed). * Correlation is significant at the 0.05 level (2-tailed).

**Table 9:** Values of *P_i_*, *I_geo_*, *E_i_*, and *RI*.

	*P_i_*	*I_geo_*	*E_i_*	*RI*
Cr	0.45	−0.47	2.16	91.59
Ni	0.36	−0.70	4.62	
Cu	0.39	−0.80	4.29	
Zn	0.26	−0.88	0.82	
As	0.16	−1.01	7.44	
Cd	0.34	−0.94	23.46	
Hg	0.07	−0.47	43.37	
Pb	0.28	−0.46	5.43	

## Data Availability

The data presented in this study are available on request from the corresponding author. The data are not publicly available because the project team do not allow us to publish the data of this study.
